# Myelin Oligodendrocyte Glycoprotein Antibody Associated Cerebral Cortical Encephalitis: Case Reports and Review of Literature

**DOI:** 10.3389/fnhum.2021.782490

**Published:** 2022-01-03

**Authors:** Hang Shu, Manqiu Ding, Pei Shang, Jia Song, Yue Lang, Li Cui

**Affiliations:** ^1^Department of Neurology, The First Hospital of Jilin University, Changchun, China; ^2^Department of Molecular Pharmacology and Experimental Therapeutics, Mayo Clinic, College of Medicine, Rochester, MN, United States

**Keywords:** cortical, encephalitis, autoimmune, seizures, MOG

## Abstract

Myelin oligodendrocyte glycoprotein antibody-associated disease is an immune-mediated demyelinating disease of the central nervous system that is present in both adults and children. The most common clinical manifestations are optic neuritis, myelitis, acute disseminated encephalomyelitis, and brainstem syndrome. Cerebral cortical encephalitis (CCE) is a rare clinical phenotype of myelin oligodendrocyte glycoprotein (MOG) antibody-associated disease (MOGAD), which usually begins with seizures, headaches, and fever, and may be misdiagnosed as viral encephalitis in the early stages. Herein, we report two typical MOG antibody (MOG-Ab)-positive patients presenting with CCE, both of whom presented with headache, fever, seizures, and who recovered completely after immunotherapy. In addition, we performed a systematic review of the present literature from the perspectives of population characteristics, clinical symptoms, MRI abnormalities, treatments, and prognosis. Among the patients reported in 25 articles, 33 met our inclusion criteria, with the age of onset ranging from 4 to 52 years. Most of the patients had seizures, headache, fever, and unilateral cortical lesions on brain MRI. For acute CCE, 30 patients were treated with high-dose intravenous methylprednisolone, and the symptoms of most patients were completely relieved after immunotherapy. This study reported our experience and lessons learned in the diagnosis and treatment of MOG-Ab-positive CCE and provides a systematic review of the literature to analyse this rare clinical phenotype.

## Introduction

Myelin oligodendrocyte glycoprotein is a membrane protein uniquely expressed on the surface of oligodendrocytes and myelin in the central nervous system of humans and other mammals (Ramanathan et al., 2016; Jain et al., 2021). Myelin oligodendrocyte glycoprotein (MOG) antibody-associated disease (MOGAD) overlaps with multiple sclerosis, acute disseminated encephalomyelitis, and aquaporin 4 antibody (AQP4-Ab)-positive neuromyelitis optica spectrum disorders (NMOSD) in terms of clinical phenotype, and is now considered to be a new and independent entity in inflammatory demyelinating diseases of the central nervous system (Ramanathan et al., 2016; Cobo-Calvo et al., 2019). The demographic, clinical, and laboratory differences at the onset of MOGAD are usually age-related. Among children, the female-to-male ratio is similar (Jurynczyk et al., 2017; Cobo-Calvo et al., 2021), and presentation at onset is usually acute disseminated encephalomyelitis, especially under 10 years of age, followed by optic neuritis (ON), transverse myelitis (TM), and brainstem demyelination (Fernandez-Carbonell et al., 2016; Duignan et al., 2018). The overall prognosis is better in children than in adults, with less than 10% of motor disability and visual acuity disability after treatment, and a lower risk of relapse in children (Reindl and Waters, 2019; Cobo-Calvo et al., 2021). Compared with children, there are slightly more female patients among adults (Jurynczyk et al., 2017), and the first presentation is usually ON (as high as 50–70%) (Duignan et al., 2018). In acute attacks, high-dose intravenous methylprednisolone is used in both adults and children, and plasma exchange is preferred when recovery is incomplete. During maintenance therapy, intravenous immunoglobulin (IVIg) is the preferred first-line treatment for children, whereas azathioprine (AZA), mycophenolate mofetil (MMF), and rituximab (RTX) are the first-line treatments for adults (Whittam et al., 2020). Some studies have reported that the higher the antibody titre at the time of onset and the longer the duration of antibody positivity, the greater the possibility of relapse (Hennes et al., 2017; Jurynczyk et al., 2017). Some patients relapsed during or after the withdrawal of steroids, most of whom showed ON (Reindl and Waters, 2019); therefore, a previous study suggested that a prolonged steroid taper can reduce early relapse of MOGAD (Narayan et al., 2018).

Since MOG antibody (MOG-Ab)-related cerebral cortical encephalitis (CCE) was first reported by Ogawa et al. (2017), many cases of this rare clinical phenotype have been reported globally, which may have been diagnosed with unexplained steroid-responsive encephalitis in the early stages of the disease (Wang et al., 2021). CCE is a syndrome with an unclear clinical definition and is characterised by grey matter lesions on brain MRI, primarily involving the cerebral cortex and sulcus, but not the subcortical and deep white matter (Krupp et al., 2013; Ogawa et al., 2017; Hamid et al., 2018). In addition to fever, headache, and seizures, cerebral cortical symptoms, such as aphasia, dysarthria, paralysis, mental symptoms, and memory loss, also exist in patients with MOG-Ab-related CCE. A typical imaging feature is the hyperintensity of cortical lesions in fluid-attenuated inversion recovery (FLAIR). According to the above imaging characteristics, CCE is divided into two types: unilateral and bilateral. In this study, we report two cases of CCE with positive MOG-Ab and perform a systematic analysis of previously reported cases. The purpose of this study was to further describe the clinical features, imaging results, and prognosis of rare MOG-Ab-positive CCE.

## Case Presentation

### Patient 1

**FIGURE 1:**
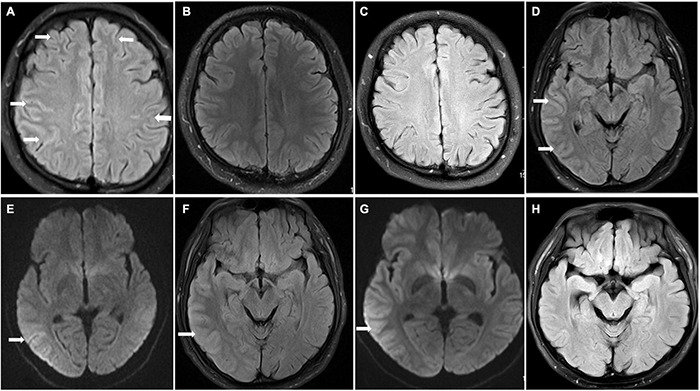
Brain magnetic resonance imaging (MRI) findings in patients 1 and 2. Patient 1 **(A–C)** On admission, FLAIR hyperintensity was seen in the leptomeningeal of bilateral frontal lobe and parietal lobe in patient 1 **(A)** (arrowhead); repeat brain MRI FLAIR result returned to normal at discharge and 8 months after discharge **(B,C)**. Patient 2 **(D–G)** Brain MRI results showed T2 FLAIR and DWI hyperintensity **(D,E)** (arrowhead) with swelling of brain tissue and narrowing of sulcus; the swelling of brain tissue was alleviated, and hyperintensity of FLAIR and DWI was improved after immunotherapy **(F,G)** (arrowhead); brain MRI normalized at 1 month after discharge **(H)**.

A previously healthy 24-year-old man presented with 2 days of fever and headache; he was diagnosed with viral encephalitis in another hospital a month ago because of “paroxysmal unconsciousness and convulsions,” which improved after antiviral and antiepileptic treatment. At the time of admission, his physical examination results were as follows: his muscle strength of the extremities was at stage V, and the pain sensation on the right side of his face was diminished. The brain MRI results showed FLAIR (Figure 1A) hyperintensity in the leptomeningeal areas of the bilateral frontal and parietal lobes without enhancement. Electroencephalogram (EEG) showed irregular sharp waves, and sharp slow waves in the left temporal region and right frontal region during the interictal period, which indicated focal discharge. The cerebrospinal fluid (CSF) displayed an elevated intracranial pressure (ICP, 295 mmH_2_O), white blood cell count (WBC, 436 × 10^6^/l), and protein (1.16 g/l) levels. The titres of MOG-Ab in CSF and serum were 1:10 and 1:100, respectively. Extensive screening for infection (CSF culture, X-pert, and Torch virus) and systemic autoimmune conditions were all negative. Therefore, the patient was diagnosed with CCE with positive MOG-Ab (Supplementary Figure 1, clinical evolution of patient 1).

The patient received immunoglobulin (0.4 g/Kg/d) for 5 days and methylprednisolone for 15 days followed by oral prednisolone (PSL). His symptoms were completely relieved after immunotherapy, and a repeat brain MRI was normal (Figure 1B). Considering the patient’s economic conditions, he was not treated with MMF, RTX, or AZA after discharge. Follow-up continued for 8 months, and no further relapse was recorded, and a repeat brain MRI showed normal results (Figure 1C).

### Patient 2

A 25-year-old man presented with headache, fever, seizures, and dysarthria without previous medical, family, or genetic history. Physical examination on admission showed that the muscle strength of the left upper limb was grade V, and the bilateral pathological signs were positive. Brain MRI results showed FLAIR (Figure 1D) hyperintensity and diffusion-weighted images (DWI; Figure 1E) showed diffusion restriction in the right temporal lobe, parietal lobe, and occipital lobe without enhancement (Supplementary Figure 2, clinical evolution of the patient 2).

After admission, he was treated with latamoxef, penciclovir, and levetiracetam. CSF analysis revealed a WBC count of 219 × 10^6^/L and elevated protein levels of 0.68 g/l. CSF autoimmune encephalopathy antibody testing showed negative results, including anti-*N*-methyl-D-aspartate receptor (NMDAR) IgG, leucine-rich glioma inactivated 1 IgG, and contactin-associated protein 2 IgG. Therefore, we considered the diagnosis of viral encephalitis and continued the treatment. However, the patient’s symptoms worsened with acute type II respiratory failure, which was treated with tracheal intubation, followed by a decrease in the left upper limb muscle strength (grade 0) on the ninth day of admission. Lumbar puncture was performed again, showing that the MOG-Ab was positive in both the CSF and serum, with a titre of 1:10 in CSF and 1:100 in serum. The patient received high-dose intravenous methylprednisolone and IVIg, which was followed by complete resolution of his symptoms. Repeat brain MRI results showed lower hyperintensity of FLAIR (Figure 1F) and DWI (Figure 1G) than before. His dose of PSL was gradually decreased after discharge. During the follow-up, repeated brain MRI normalised at 1 month without relapse (Figure 1H).

## Materials and Methods

**FIGURE 2:**
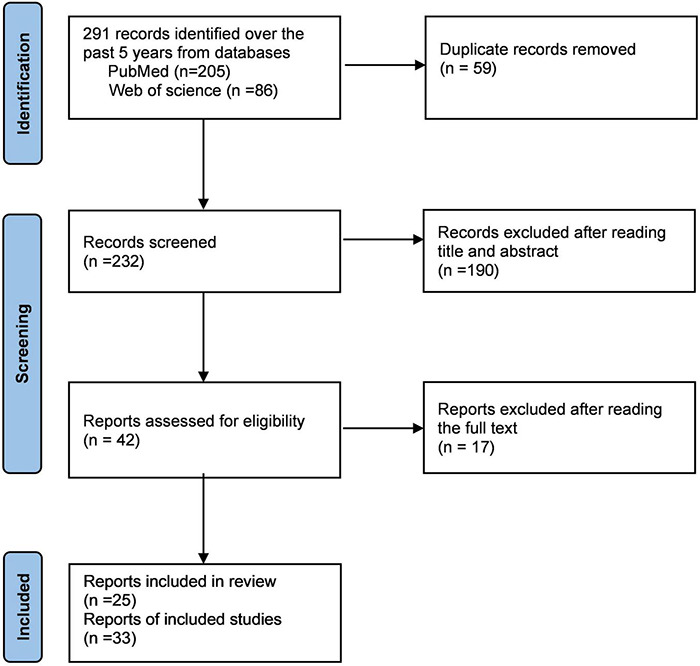
Review flow-chart.

To obtain a more thorough understanding of MOG-Ab-associated CCE, we searched and screened studies in PubMed and Web of Science until August 2021 to identify patients according to the following process (Figure 2, review flowchart). The inclusion criteria were as follows: (1) Patients who tested positive only for serum MOG-Ab during the course of the disease, and no other antibodies such as AQP4-Ab, NMDAR-Ab, and contactin-associated protein 2-Ab that may indicate other diseases. (2) Patients explicitly diagnosed with MOG-Ab-related CCE for the first time. (3) Presence of abnormal hyperintensity on brain MRI, mainly in the cerebral cortex or sulcus. (4) Availability of complete data on clinical symptoms, imaging, and treatment.

## Results

We finally obtained 33 cases from 25 articles and combined with our two cases, a total of 35 cases were reviewed systematically (Ogawa et al., 2017; Hamid et al., 2018; Ikeda et al., 2018; Budhram et al., 2019, 2020; Haddad et al., 2019; Patterson et al., 2019; Fujimori et al., 2020b; Hochmeister et al., 2020; Katsuse et al., 2020; Matoba et al., 2020; Otani et al., 2020; Russ et al., 2020; Takamatsu et al., 2020; Tao et al., 2020; Ahsan et al., 2021; Chang et al., 2021; Doig et al., 2021; Jain et al., 2021; Kim et al., 2021; Maniscalco et al., 2021; Nie et al., 2021; Stamenova et al., 2021; Tian et al., 2021; Wang et al., 2021; Table 1). The main demographic data, clinical characteristics, laboratory results, and imaging results are presented in Table 2.

**TABLE 1:** Summary of MOG-Ab associated cerebral cortical encephalitis cases included in this study.

Case	Author/Year	Demographic statistics	Clinical symptoms				Imaging	Serum	Immunotherapy		Relapse	Outcome
		Age/Sex	Headache	Fever	Seizure	Paralysis	Lesion	MOG-Ab titer	HIMP	Other therapy		
1	Jain/2021	31/F	Yes	No	Yes	No	Unilateral cortical	1:10	Yes	MM	No	Full recovery
2	Ogawa/2017	23/M	Yes	No	Yes	No	Unilateral cortical	1:256	No	No	No	Full recovery
3	Ogawa/2017	38/M	No	No	Yes	Yes	Unilateral cortical	1:1024	Yes	No	No	Full recovery
4	Ogawa/2017	38/M	No	No	Yes	Yes	Unilateral cortical	1:512	Yes	No	No	Full recovery
5	Ogawa/2017	36/M	No	No	Yes	No	Unilateral cortical	1:2048	Yes	No	No	Full recovery
6	Hamid/2018	Teens/M	Yes	No	Yes	No	Unilateral cortical	NR	Yes	No	Yes	Improved
7	Tao/2020	28/M	Yes	Yes	Yes	No	Unilateral cortical	1:10	Yes	No	No	Full recovery
8	Tao/2020	32/M	Yes	Yes	Yes	No	Unilateral cortical	1:10	Yes	MM	No	Full recovery
9	Katsuse/2020	44/F	No	No	No	No	Unilateral cortical	1:128	Yes	No	No	Full recovery
10	Otani/2020	22/F	Yes	Yes	Yes	No	Unilateral cortical	1:1024	No	No	No	Full recovery
11	Budhram/2020	21/F	Yes	No	Yes	No	Unilateral cortical	1:80	No	No	No	Improved
12	Russ/2020	11/F	No	Yes	Yes	Yes	Unilateral cortical	NR	Yes	RTX	Yes	Improved
13	Tian/2021	8/F	Yes	Yes	N0	No	Unilateral cortical	1:10	Yes	No	No	Full recovery
14	Tian/2021	10/F	Yes	No	Yes	Yes	Unilateral cortical	1:10	Yes	No	No	Full recovery
15	Budhram/2019	23/M	Yes	Yes	Yes	Yes	Unilateral cortical	NR	Yes	No	No	Full recovery
16	Fujimori/2020	31/M	Yes	No	No	No	Unilateral cortical	1:1024	Yes	No	No	Full recovery
17	Patterson/2019	39/F	Yes	Yes	No	No	Unilateral cortical	NR	Yes	CTX	No	Improved
18	Maniscalco/2021	18/F	No	No	Yes	Yes	Unilateral cortical	1:2560	Yes	RTX	No	Full recovery
19	Ahsan/2021	7/F	Yes	No	Yes	Yes	Unilateral cortical	1:40	No	IVIg	No	Improved
20	Nie/2021	19/F	Yes	Yes	Yes	No	Unilateral cortical	1:512	Yes	No	No	Full recovery
21	Chang/2021	34/M	Yes	No	Yes	No	Unilateral cortical	NR	Yes	No	No	Full recovery
22	Matoba/2020	29/F	Yes	No	No	Yes	Unilateral cortical	NR	Yes	No	No	Full recovery
23	Doig/2020	11/M	Yes	Yes	Yes	No	Bilateral cortical	NR	Yes	IVIg	No	Improved
24	Doig/2020	4/M	Yes	Yes	Yes	Yes	Unilateral cortical	NR	Yes	IVIg and PE	No	Improved
25	Takamatsu/2020	15/M	No	Yes	Yes	No	Unilateral cortical	NR	No	No	No	Improved
26	Kim/2020	44/M	No	Yes	No	No	Unilateral cortical	NR	Yes	No	No	Full recovery
27	Kim/2020	52/F	Yes	No	Yes	No	Unilateral cortical	NR	Yes	AZA	No	Full recovery
28	Haddad/2019	23/M	Yes	No	Yes	Yes	Unilateral cortical	1:1000	Yes	No	No	Full recovery
29	Stamenova/2021	31/F	Yes	Yes	Yes	No	Bilateral cortical	NR	Yes	AZA	No	Improved
30	Wang/2021	19/M	Yes	Yes	Yes	No	Unilateral cortical	1:32	Yes	MM	No	Full recovery
31	Wang/2021	23/M	Yes	No	Yes	No	Unilateral cortical	1:32	Yes	MM	No	Full recovery
32	Hochmeister/2020	52/F	Yes	No	No	No	Bilateral cortical	1:320	Yes	No	Death	Death
33	Ikeda/2018	29/F	No	Yes	Yes	No	Bilateral cortical	1:1024	Yes	No	No	Full recovery
34	patient 1	24/M	Yes	Yes	Yes	No	Bilateral cortical	1:100	Yes	IVIg	No	Full recovery
35	patient 2	25/M	Yes	Yes	Yes	Yes	Unilateral cortical	1:100	Yes	IVIg	No	Full recovery

*F, female; M, male; MOG-Ab, myelin oligodendrocyte glycoprotein antibody; NR, not reported; HIMP, high-dose intravenous methylprednisolone; IVIg, intravenous immunoglobulin; PE, plasma exchange; RTX, rituximab; MM, mycophenolate mofetil; AZA, azathioprine; CTX, cyclophosphamide.*

**TABLE 2:** Main demographic data, clinical characteristics, laboratory, and imaging results in described MOG-Ab associated cerebral cortical encephalitis.

Population characteristics	
Mean age, years	25 (Range 4–52)
Median age, years	24
Sex	19M/16F (M: F = 19:16)
**Clinical presentation**	
Seizures	28/35 (80.0%)
Headache	26/35 (74.3%)
Fever	17/35 (48.6%)
Paralysis	11/35 (31.4%)
Psychiatric symptoms	10/35 (28.6%)
Language disorder	9/35 (25.7%)
**Antibody test (CBA)**	
Serum Mog-IgG	35/35 (100%)
Mog-IgG titer	1:10–1:2560
EEG patterns	
Slow wave	8/18 (44.4%)
Epileptiform wave	5/18 (27.8%)
Normal	3/18 (16.7%)
**Neuroimaging**	
FLAIR hyperintensity	31/35 (88.6%)
Unilateral cortical involvement	30/35 (85.7%)
Bilateral cortical involvement	5/35 (14.3%)
**Cortical involvement site**	
Parietal lobe	24/35 (68.6%)
Frontal lobe	24/35 (68.6%)
Temporal lobe	15/35 (42.9%)
Occipital lobe	6/35 (17.1%)
**Intervention**	
HIMP	30/35 (85.7%)
IVIg	5/35 (14.3%)
Immunosuppressant	9/35 (25.7%)
Relapse	2/35 (5.7%)
**Outcome**	
Fully recovery	25/35 (71.4%)
Partial recovery	9/35 (25.7%)
Death	1/35 (2.9%)

*M, male; F, female; CBA, cell-based assay; MOG, myelin oligodendrocyte glycoprotein; EEG, electroencephalogram; FLAIR, fluid-attenuated inversion recovery; HIMP, high-dose intravenous methylprednisolone; IVIg, intravenous immunoglobulin.*

### Demographic Data

Among the 35 patients, 16 were women and 10 were men. The average age of onset of the patients was 25 years (range 4–52 years), and the median age for disease onset was 24 years.

### Clinical Symptoms

The clinical symptoms of the disease included seizures, headache, fever, paralysis, mental disorders, language disorders, disorientation, memory decline, and autonomic nervous system dysfunction. The most prominent clinical manifestation was a seizure, which occurred in 28 (82.6%) patients during the course of the disease. Among the 28 cases with seizures, 23 described the types of seizures: 19 patients presented with generalised tonic-clonic seizures (GTCS) at the time of onset, and seven patients initially presented with focal motor seizures, three of whom eventually developed GTCS.

### Magnetic Resonance Imaging Patterns

The study found that 21 patients showed cortical hyperintensity on the MRI FLAIR sequence, while the others showed swelling of the cortex and narrowing or disappearance of the sulcus. Thirty patients showed unilateral cortical involvement, of which the frontal lobe and parietal lobe were most likely to be involved, while the occipital lobe was rarely involved. Of the five patients with bilateral CCE, four had bilateral frontal lobes involved. Twenty-one patients received brain MRI DWI sequence in the acute setting, nine of whom had diffusion limitation.

### Myelin Oligodendrocyte Glycoprotein Antibody Titre

Serum MOG-Abs were detected by a cell-based assay in all patients at the onset or during the course of the disease. The titres of MOG-Abs vary greatly, ranging from 1:10 to 1:2,560. In general, a higher titre value results in a longer duration of headache and fever, a higher frequency of seizures, and a greater degree of visual impairment and paralysis. After immunotherapy, the antibody titre decreased significantly and could even be negative.

### Electroencephalogram Patterns

Since most patients exhibit seizures as a first symptom, EEG is necessary for differential diagnosis and further treatment. Eighteen patients underwent EEG, and 15 presented with abnormal EEG, which can present as slow waves, epilepsy waves, or other abnormalities.

### Treatments

In the acute setting of CCE, 30 patients received high-dose intravenous methylprednisolone, four of whom received it combined with IVIg. During the remission period of maintenance therapy, nine patients received immunosuppressants to prevent relapse, including MMF, RTX, AZA, and cyclophosphamide.

### Prognosis

Among all the patients, 25 recovered completely, as depicted by both radiodiagnosis and symptoms. Additionally, nine partially recovered, and one died. During the follow-up period, there were two patients with relapse, one presented with ON, and the other presented with headache and seizures, both of whom partially recovered eventually.

## Discussion

Compared with the previously reported CCE, our cases were generally consistent with the previous cases in terms of clinical manifestation, imaging, and treatment. However, the rapid deterioration of patient 2 emphasizes the importance and necessity of early diagnosis and treatment. MOG-Ab-associated CCE may produce severe clinical symptoms, and even deaths have been reported. Therefore, early diagnosis and treatment are needed, which will greatly improve the prognosis of patients; otherwise, serious complications may occur. Since the most common symptoms are seizures, headache, and fever, it is easily misdiagnosed as intracranial infection, such as viral encephalitis and meningeal inflammation (Budhram et al., 2019). At present, as a new method for the diagnosis of pathogen infection, metagenomic next-generation sequencing can help improve the detection rate of encephalitis caused by pathogen infection (Wilson et al., 2019).

Although our study did not include cortical encephalitis caused by other pathogens, during the screening of the literature we found that MOG-Ab can coexist with other antibodies, especially anti-NMDAR-Ab (Zhou et al., 2017; Sinani et al., 2020; Nan et al., 2021). The new data show that NMDARs exist on the myelin sheath formed by oligodendrocytes (Lipton, 2006); therefore, NMDAR may be positive and can cause demyelination when an immune response occurs. In an analysis of 691 cases of anti-NMDAR encephalitis, 12 cases were found to be MOG-Ab-positive at the same time (Titulaer et al., 2014). When a patient has symptoms of CCE, it is difficult to identify whether it is caused by MOG-Ab or anti-NMDAR-Ab. Fortunately, their treatment principles are generally similar, consisting of HIMP or IVIg. Notably, RTX is more likely to be used to treat anti-NMDAR encephalitis with an acute onset (Dalmau et al., 2011; Dou et al., 2020; Thaler et al., 2021), but only in remission periods for maintenance treatment in MOGAD. We recommend that patients with MOGAD should be tracked and simultaneously tested for MOG-Ab and other antibodies in the serum and CSF for the early detection of an overlapping syndrome.

In a single-centre cohort study from China, 20.7% of MOG-Ab-positive patients had typical encephalitis symptoms, and 72.2% had cortical changes during the course of encephalitis (Wang et al., 2019). Seizures are the most common symptom of CCE, which may be caused by immune-mediated demyelination. Immunotherapy may be more effective than conventional antiepileptic therapy (Cobo-Calvo et al., 2018). In general, cortical damage caused by epilepsy is characterised by hyperintensity on DWI, which indicates cytotoxic oedema (Milligan et al., 2009; Chatzikonstantinou et al., 2011), while cortical damage caused by MOG-Ab-related CCE is usually indicated by abnormal hyperintensity on FLAIR. “FLAMES” is a new term used to characterise the clinical and radiological syndromes of cortical FLAIR-hyperintense lesions in anti-MOG-Ab-associated encephalitis with seizures (Budhram et al., 2019). A study has pointed out that long-term oral anti-epileptic drugs (AEDs) have no significant effect on the control of seizures, while immunosuppressant MMF can significantly reduce the occurrence of acute seizures (Yao et al., 2019). In our systematic review, since most patients received steroid therapy and AEDs at the same time, the effect of AEDs alone was unknown. Therefore, it is still not clear whether AEDs should be used for extended periods, and it should be evaluated comprehensively according to the effect of steroid application, along with the type, duration, and frequency of seizures.

In our study, 31 patients showed cortical hyperintensity on brain MRI FLAIR, which was relatively unique but not specific. In the case of atypical symptoms, they should be distinguished from other cortical diseases, such as seizures, Creutzfeldt-Jakob disease, subarachnoid haemorrhage, meningeal carcinoma, and reversible posterior leukoencephalopathy syndrome (Renard et al., 2015). Several researchers have reported seizure-induced reversible MRI abnormalities (SRMA) that are characterised by MRI abnormalities, which resolve completely at follow-up (Cole, 2004; Briellmann et al., 2005). In a systematic review from the University of Melbourne in Australia, which involved 27 patients with SRMA, 12 (44%) patients demonstrated SRMA in the cortical layer on brain MRI and 21 (78%) patients showed hyperintensity on MRI FLAIR (Mariajoseph et al., 2021). Based on the similarity between SRMA and MOG-Ab-mediated CCE, the detection of MOG antibodies is vital for accurate diagnosis.

According to the results of our review, involvement of the cortex was mostly limited to the unilateral cerebral cortex. In a systematic review, brain MRI lesions with bilateral medial and frontal cerebral cortical encephalitis were superimposed with a computer software program, showing that most of the lesions were distributed in the supply area of the anterior cerebral artery, while the lesions with unilateral frontal cerebral cortical encephalitis were distributed in the middle cerebral artery region (Fujimori et al., 2020a). Therefore, we speculated that the lesions of the cortex are involved in vascular distribution. Reviewing the cases in our study, we observed that in the 11 patients who underwent cerebrovascular perfusion tests, including MRI, arterial spin labelling imaging, and single-photon emission computed tomography, 10 patients displayed vasodilation of the cerebrovascular branch, indicating high cerebrovascular perfusion (Ogawa et al., 2017; Ikeda et al., 2018; Fujimori et al., 2020b; Katsuse et al., 2020; Matoba et al., 2020; Otani et al., 2020; Jain et al., 2021; Nie et al., 2021), which usually can be completely restored to normal after steroid therapy. Although there is no clear conclusion about the relationship between vascular distribution and lesions, we speculate that peripheral vessels of the middle cerebral artery or anterior cerebral artery may also be involved in the pathogenesis of the disease after cortical inflammation occurs.

It was found that the titre value of MOG-Ab in the serum of the patients varied greatly, which can be used to predict relapse (Tea et al., 2019; Marchionatti et al., 2021), supporting the pathogenic role of MOG-Ab. However, the MOG-Ab titre value was not entirely positively correlated with the severity and recurrence of the patient’s disease in our study, which may be related to the timing of serum collection, meaning that the titre value will decrease significantly after steroid therapy. In a patient with case 30, although the patient’s MOG-Ab titre was 1:320, it manifested itself with fulminant phenotype, and the patient died shortly due to older age and extensive cortical involvement. Therefore, for each individual, we should make a comprehensive judgment of the patient’s prognosis according to age, past medical history, and clinical characteristics.

The first-line treatment of patients includes HIMP and IVIg in the acute setting, and significant improvements in clinical symptoms and imaging can usually be observed after steroid treatment. By reviewing the patients’ prognosis, we found that the lesions resolved completely in most patients, which is significantly different from that in multiple sclerosis and NMOSD; the underlying mechanisms of which are not yet fully understood. In a retrospective cohort study from the Mayo Clinic, comparing the evolution of MRI lesions in different central nervous system demyelinating disorders, MRI normalisation of brain T2 signal abnormality was more common with MOGAD for brain attacks (MOGAD, 39%; AQP4-IgG-NMOSD, 10%; Multiple sclerosis, 5%), and the greater remyelination capacity, less neuronal loss, or a predominant functional vs. structural damage in MOGAD might account for this difference (Sechi et al., 2021).

However, some patients relapse after withdrawal therapy (Cobo-Calvo et al., 2019), and therefore, patients may be treated with immunosuppressive therapy, such as MMF, RTX, and cyclophosphamide, to prevent relapse. The patient may relapse with CCE again, or any type of MOGAD. Although the MOG-Ab titre value can be used to predict relapse, it cannot be relied upon alone. New research suggests that FNFAIP3 levels decreased at relapse compared to remission, which has clinical utility as a relapse biomarker and potential therapeutic target (Saxena et al., 2020). Steps are being undertaken toward its potential use in clinical settings.

Our study was retrospective and had certain limitations. Currently, MOG-Ab-associated CCE is still rare, the sample size is not large enough, and the results may be biased. Moreover, the follow-up time of each case varied, which may have affected the prognosis.

## Conclusion

In conclusion, MOG-Ab-associated CCE is still a rare clinical type of MOGAD. In patients with headaches, fever, and seizures, the detection of serum MOG-Ab should be performed at the earliest, in order to make a clear diagnosis and administer timely treatment. Moreover, the regular detection of MOG-Ab titres during follow-up contributes to the early detection of disease relapse.

## Data Availability Statement

The original contributions presented in the study are included in the article/Supplementary Material, further inquiries can be directed to the corresponding author.

## Ethics Statement

The studies involving human participants were approved by the Human and Research Ethics committees of the first Bethune Hospital of Jilin University. The patients/participants provided written informed consent for the publication of any identifiable data/images presented in the manuscript.

## Author Contributions

MD and HS conceived the topic, designed the outline of this review, and wrote the manuscript. YL and JS prepared the figures and tables. LC and PS critically revised the manuscript. All authors contributed to the article and approved the submitted version.

## Conflict of Interest

The authors declare that the research was conducted in the absence of any commercial or financial relationships that could be construed as a potential conflict of interest.

## Publisher’s Note

All claims expressed in this article are solely those of the authors and do not necessarily represent those of their affiliated organizations, or those of the publisher, the editors and the reviewers. Any product that may be evaluated in this article, or claim that may be made by its manufacturer, is not guaranteed or endorsed by the publisher.

## Abbreviations

AED: anti-epileptic drugs
AQP4-Ab: aquaporin 4 antibody
CCE: cerebral cortical encephalitis
DWI: diffusion-weighted images
EEG: electroencephalogram
FLAIR: fluid-attenuated inversion recovery
MOG: myelin oligodendrocyte glycoprotein
MOGAD: myelin-associated disease
MOG-Ab: myelin oligodendrocyte glycoprotein antibody
NMOSD: neuromyelitis optica spectrum disorders
IVIg: intravenous immunoglobulin
MMF: mycophenolate mofetil
RTX: rituximab
NMDR: anti-*N*-methyl-D-aspartate receptor
SRMA: seizure-induced reversible MRI abnormalities.
