# Supplementary figures and images for: Myelin Oligodendrocyte Glycoprotein Antibody Associated Cerebral Cortical Encephalitis: Case Reports and Review of Literature
Source: Front Hum Neurosci. 2022 Jan 3;15:782490. doi: 10.3389/fnhum.2021.782490 (PMC8762331; doi:10.3389/fnhum.2021.782490)

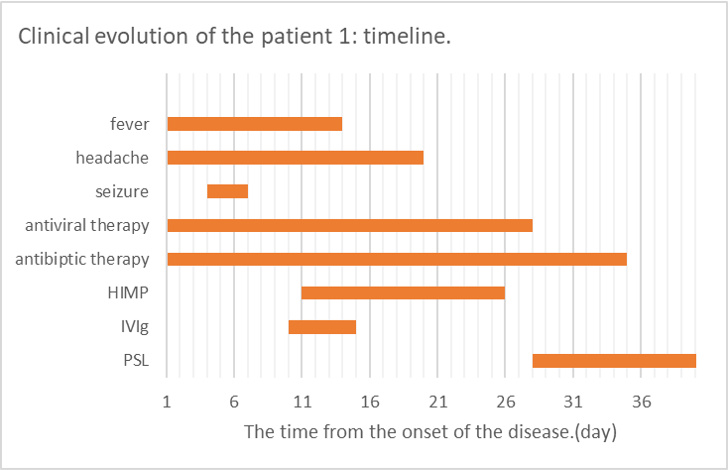

Supplement: Supplementary file 1 [file Image_1.JPEG]

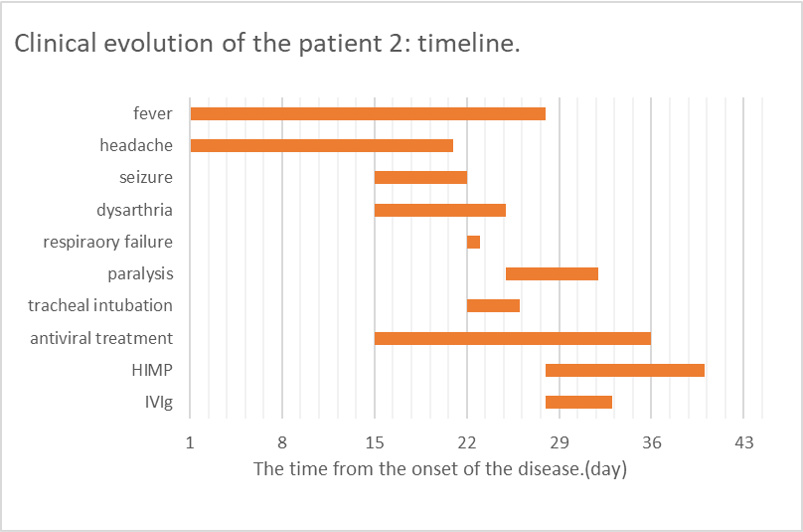

Supplement: Supplementary file 2 [file Image_2.JPEG]
